# Three steps in one pot: biosynthesis of 4-hydroxycinnamyl alcohols using immobilized whole cells of two genetically engineered *Escherichia coli* strains

**DOI:** 10.1186/s12934-017-0722-9

**Published:** 2017-06-12

**Authors:** Shuxin Liu, Jiabin Liu, Jiayin Hou, Nan Chao, Ying Gai, Xiangning Jiang

**Affiliations:** 1 College of Biological Science and Technology, Beijing Forestry University, Beijing, 100083 People’s Republic of China; 2 The Tree and Ornamental Plant Breeding and Biotechnology Laboratory of Chinese Forestry Administration, National Engineering Laboratory for Tree Breeding, Beijing, 100083 People’s Republic of China

**Keywords:** 4**-**Hydroxycinnamyl alcohols, 4-Coumaric acid: coenzyme A ligase, Cinnamoyl coenzyme A reductase, Cinnamyl alcohol dehydrogenase, Immobilized whole-cell, *Escherichia coli*

## Abstract

**Background:**

4-Hydroxycinnamyl alcohols are a class of natural plant secondary metabolites that include *p*-coumaryl alcohol, caffeyl alcohol, coniferyl alcohol and sinapyl alcohol, and have physiological, ecological and biomedical significance. While it is necessary to investigate the biological pathways and economic value of these alcohols, research is hindered because of their limited availability and high cost. Traditionally, these alcohols are obtained by chemical synthesis and plant extraction. However, synthesis by biotransformation with immobilized microorganisms is of great interest because it is environmentally friendly and offers high stability and regenerable cofactors. Therefore, we produced 4-hydroxycinnamyl alcohols using immobilized whole cells of engineered *Escherichia coli* as the biocatalyst.

**Results:**

In this study, we used the recombinant *E. coli* strain, M15–4CL1–CCR, expressing the fusion protein 4-coumaric acid: coenzyme A ligase and the cinnamoyl coenzyme A reductase and a recombinant *E. coli* strain, M15–CAD, expressing cinnamyl alcohol dehydrogenase from *Populus tomentosa* (*P. tomentosa*). High performance liquid chromatography and mass spectrometry showed that the immobilized whole cells of the two recombinant *E. coli* strains could effectively convert the phenylpropanoic acids to their corresponding 4-hydroxycinnamyl alcohols. Further, the optimum buffer pH and the reaction temperature were pH 7.0 and 30 °C. Under these conditions, the molar yield of the *p*-coumaryl alcohol, the caffeyl alcohol and the coniferyl alcohol was around 58, 24 and 60%, respectively. Moreover, the highly sensitive and selective HPLC–PDA–ESI–MSn method used in this study could be applied to the identification and quantification of these aromatic polymers.

**Conclusions:**

We have developed a dual-cell immobilization system for the production of 4**-**hydroxycinnamyl alcohols from inexpensive phenylpropanoic acids. This biotransformation method is both simple and environmental-friendly, which is promising for the practical and cost effective synthesis of natural products.

**Graphical abstract:**
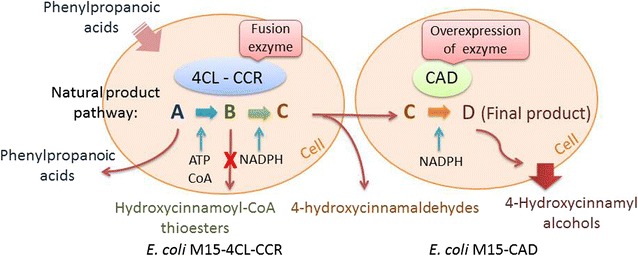
Biotransformation process of phenylpropanoic acids by immobilized whole-cells

## Background

4-Hydroxycinnamyl alcohols such as *p*-coumaryl alcohol, caffeyl alcohol, coniferyl alcohol and sinapyl alcohol are primarily produced in plants [1–3]. Because 4-hydroxycinnamyl alcohols are important intermediates in several secondary metabolism pathways, they are of considerable interest in biological chemistry, plant sciences, bioenergy research and the food industry [4–7]. These aromatic polymers are involved in the biosynthesis and degradation of lignin, which is essential for water transport, mechanical support and for plant defense against pathogens [8–10]. Research suggests that derivatives of *p*-coumaryl alcohol may serve as dietary antioxidants with important effects on immune function, and are commonly used in the food industry [11]. Gum benzoin also contains significant amounts of coniferyl alcohol, and its esters can be used as chewing gum bases and flavoring substances [12]. Moreover, these alcohols are significant and valuable in biomedical science. For example, coniferyl alcohol is the key intermediate of silibinin, a safe and effective anti-hepatitis drug [13, 14]. As such, 4-hydroxycinnamyl alcohols are of great importance for scientific research and for practical applications.

However, extensive use of these natural resources is restricted by their limited availability and high cost. In nature, these alcohols are difficult to directly extract from plants because of the large number of diverse lignin monomers and the complex and irregular structure of lignin, which result in an inefficient isolation and purification process. Because of the need for 4-hydroxycinnamyl alcohols, an effective method to artificially synthesize these compounds is necessary. Although these aromatic compounds can be synthesized by a chemical approach, previous studies found that chemical synthesis involved many complicated steps and excessive by-produces. These disadvantages are cost prohibitive, and a pure product is difficult to produce [15, 16]. Alternatively, the biotransformation method is a promising solution for the production of these aromatic polymers. Biotransformation is one of the important technical means in the field of synthetic biology, which could effectively convert relatively inexpensive and available precursors into the corresponding valuable fine chemical product [17–20].

The immobilization of microorganisms first emerged as green biocatalysts, and is becoming one of the great tools for biotransformation as a promising alternative to immobilized enzymes [21]. Immobilization means that microbial cells are physically confined or localized in a certain defined region of space while retaining their catalytic activities, and can be used repeatedly and continuously. They are of interest because of the many advantages offered by microbial cell immobilization, such as increased productivity, easy separation that allows repeated uses or continuous processes, the reduction of operational costs, mild operating conditions in terms of temperature and pH, high process stability and the protection of labile cells [21]. Immobilized microorganisms are already being used in many industrial fields, such as pharmaceuticals, food, bioenergy, biomedical science and environmental protection [22–30].

**Fig. 1:**
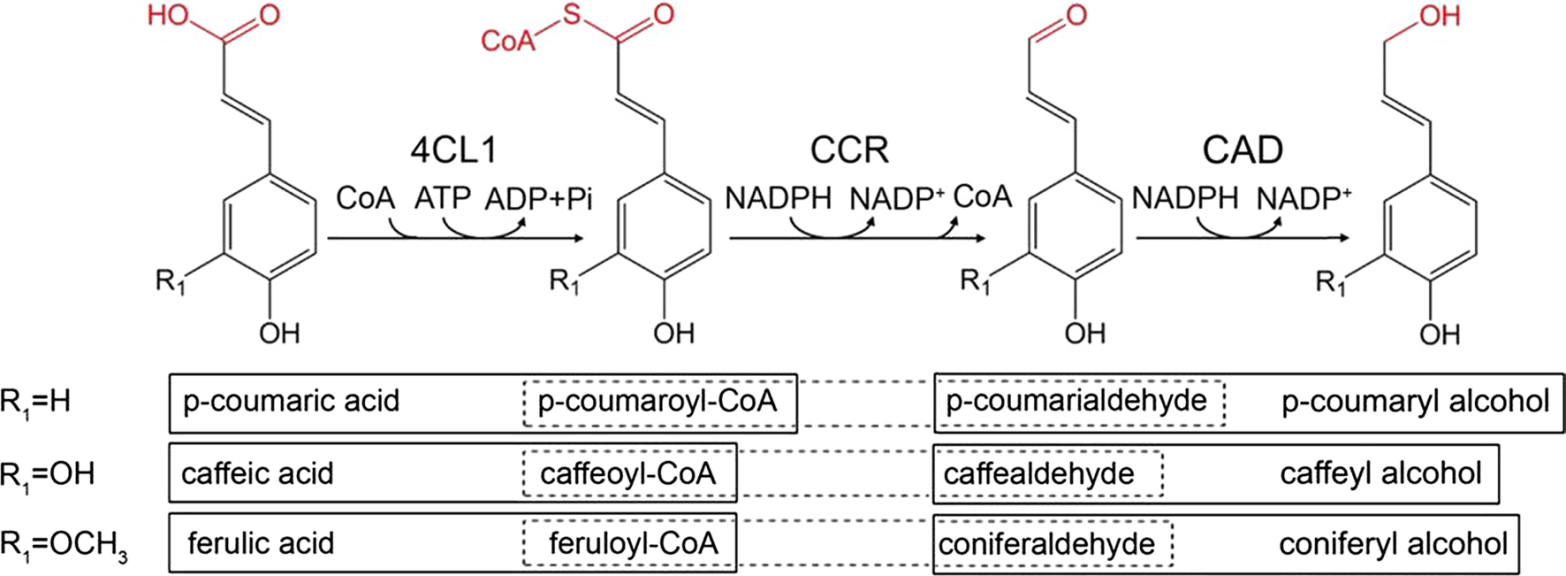
The 4-hydroxycinnamyl alcohols biosynthesis reactions catalyzed by 4-Coumaric acid: coenzyme A ligase (4CL1), cinnamoyl coenzyme A reductase (CCR) and cinnamyl alcohol dehydrogenase (CAD) from *P. tomentosa*. The prevalent conversions occurring in *P. tomentosa* are *outlined*

The formation of 4-hydroxycinnamyl alcohols is catalyzed by 4-coumaric acid: coenzyme A ligase (4CL1), cinnamoyl coenzyme A reductase (CCR) and cinnamyl alcohol dehydrogenase (CAD). Phenylpropanoic acids are activated with coenzyme A (CoA) by 4CL1 in the presence of adenosine triphosphate (ATP) and subsequently reduced by CCR and CAD to 4-hydroxycinnamyl alcohols in the presence of NADPH (Fig. 1) [31–33]. In our previous study, the genetically *Escherichia coli* strain M15–4CL1–CCR expressing the fusion protein 4CL1 and CCR was constructed for the whole-cell biotransformation system and then was successfully applied for the production of 4-hydroxycinnamaldehydes [34]. Moreover, our previous research indicated that the fusion protein 4CL1–CCR had high selectivity and could effectively catalyze the reduction of acids to aldehydes inside cells. These promising effects were believed to contribute to the whole cell’s ability to provide a natural environment for the enzyme and regenerate redox cofactors in vivo.

In this study, we developed a novel, fast and highly efficient biological technique to convert diverse phenylpropanoic acids to their corresponding 4**-**hydroxycinnamyl alcohols using immobilized whole cells of recombinant *E. coli* as the biocatalyst, along with the recombinant *E. coli* strain M15–4CL1–CCR and the recombinant *E. coli* strain M15–CAD expressing CAD from *Populus tomentosa* (*P. tomentosa*) [34, 35]. The goals of this study were: (1) to establish a rapid HPLC–PDA–ESI–MSn method for the characterization of 4**-**hydroxycinnamyl alcohols; (2) to explore the feasibility of using immobilized whole cells of two recombinant *E. coli* to catalyze the conversion; (3) to investigate the optimum buffer pH and reaction temperature to improve the production; and (4) to evaluate the productivity of this novel biosynthesis system. To the best of our knowledge, there are no reports regarding the application of immobilized whole cells for the production of 4**-**hydroxycinnamyl alcohols. This innovative system is promising for the practical and cost effective synthesis of natural products.

## Results

### Characterization of 4-hydroxycinnamyl alcohols by HPLC–PDA–ESI–MSn

To separate and identify the metabolites, a highly sensitive and selective HPLC–PDA–ESI–MSn method was established. Three kinds of phenylpropanoic acids and their corresponding 4-hydroxycinnamaldehydes and 4-hydroxycinnamyl alcohols, with internal standard substances, for a total of 10 kinds of compounds, were separated by high performance liquid chromatography (HPLC). According to the different retention times, each compound was clearly distinguishable (Table 1). The gradient elution condition was appropriate for the separation of our samples. The ultraviolet absorption wavelength was set at 340 nm for the acids and aldehydes and 280 nm for the alcohols, because acids, aldehydes and alcohols cannot be completely detected under the same wavelength (Table 1). Then, different ultraviolet absorption wavelength parameters were set to optimize the detection condition.

**Table 1:** Linear regression of phenylpropanoic acids, 4-hydrocinnaldehydes and 4-hydroxycinnamyl alcohols for quantitative analysis

Compound	Ultraviolet wavelength (nm)	Retention time (min)	Regression equation	Correlation coefficient (*R* ^2^)
*p*-Coumaric acid	340	25.41	y = 5.68E + 04x − 3.50E + 04	0.9998
Caffeic acid	340	10.28	y = 1.07E + 05x + 2.21E + 04	0.9998
Ferulic acid	340	32.40	y = 1.45E + 05x − 7.23E + 05	0.9958
*p*-Coumaraldehyde	340	34.72	–	–
Caffeldehyde	340	20.99	–	–
Coniferaldehyde	340	40.83	y = 5.43E + 05x − 9.79E + 05	0.9957
Sinapaldehyde	340	43.91	y = 3.30E + 05x − 3.86E + 05	0.9986
*p*-Coumaryl alcohol	280	16.35	–	–
Caffeyl alcohol	280	6.65	–	–
Coniferyl alcohol	280	23.24	y = 8.81E + 04x − 8.22E + 04	0.9997

Authentic standards of 6 compounds (*p*-coumaric acid, caffeic acid, ferulic acid, coniferaldehyde, sinapaldehyde and coniferyl alcohol) were serially diluted and injected into the HPLC–PDA–ESI–MSn system to obtain the calibration curves. As shown in Table 1, each compound exhibited excellent linearity, with *R* ^*2*^ values from 0.9957 to 0.9998. This result indicated a good correlation between the A340 (or A280) values and the concentrations of the standard solutions. The retention time of the *p*-coumaraldehyde, caffeldehyde, *p*-coumaryl alcohol, and caffeyl alcohol was not obtained from the standards, but rather from the analyses of our synthesized samples.

Solutions containing *p*-coumaryl alcohol, caffeyl alcohol isolated from our samples, and standard coniferyl alcohol were prepared to determine the pattern of the parent-to-fragment (precursor-to-product) ion transition of each compound under electrospray ionization ion trap mass spectrometry (ESI–Ion trap–MS). The specific ions of the precursor and the products of selected reaction monitoring (SRM) of MS2 are summarized in Table 2. Based on the signal intensities and the signal-to-noise ratios, the negative ion scan mode was chosen for the alcohols and acids, while the positive ion scan mode was chosen for the aldehydes. The collision energies were optimized to maximize the signals from the target ions. We believe that the complete mass spectrometry data (including the characteristic fragment ions) of the *p*-coumaryl alcohol, the caffeyl alcohol and the coniferyl alcohol have not been previously reported. The appropriate transitions of the precursor-to-product ions of the acids and the aldehydes are described in our previous study [34]. Therefore, this method is suitable for subsequent analysis in this study.

**Table 2:** Optimized MS(n) condition for detection of 4-hydroxycinnamyl alcohols

Compound	Scan mode	Precursor ion	Collision energy (eV)	Product ion of SRM mode (MS2)
*p*-Coumaryl alcohol	–	149	30	131
Caffeyl alcohol	–	165	30	147
Coniferyl alcohol	–	179	30	161

### Production of 4-hydroxycinnamyl alcohols in immobilized *E. coli*

**Fig. 2:**
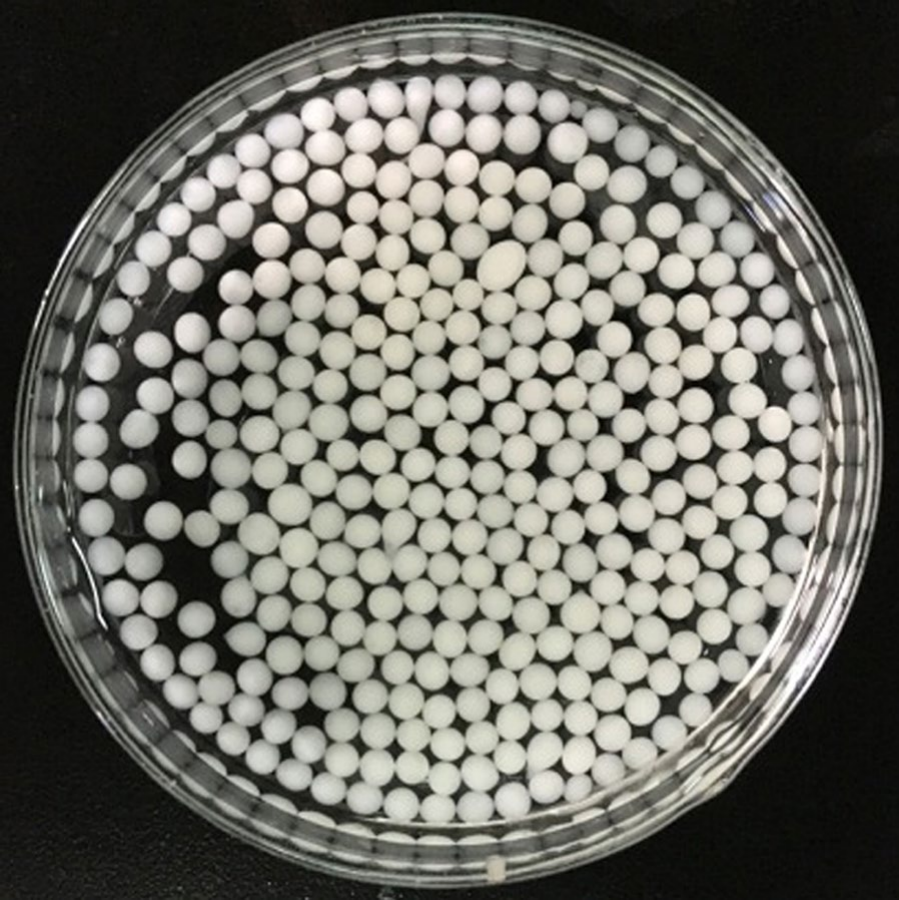
Preparation of immobilized whole cell pellets

**Fig. 3:**
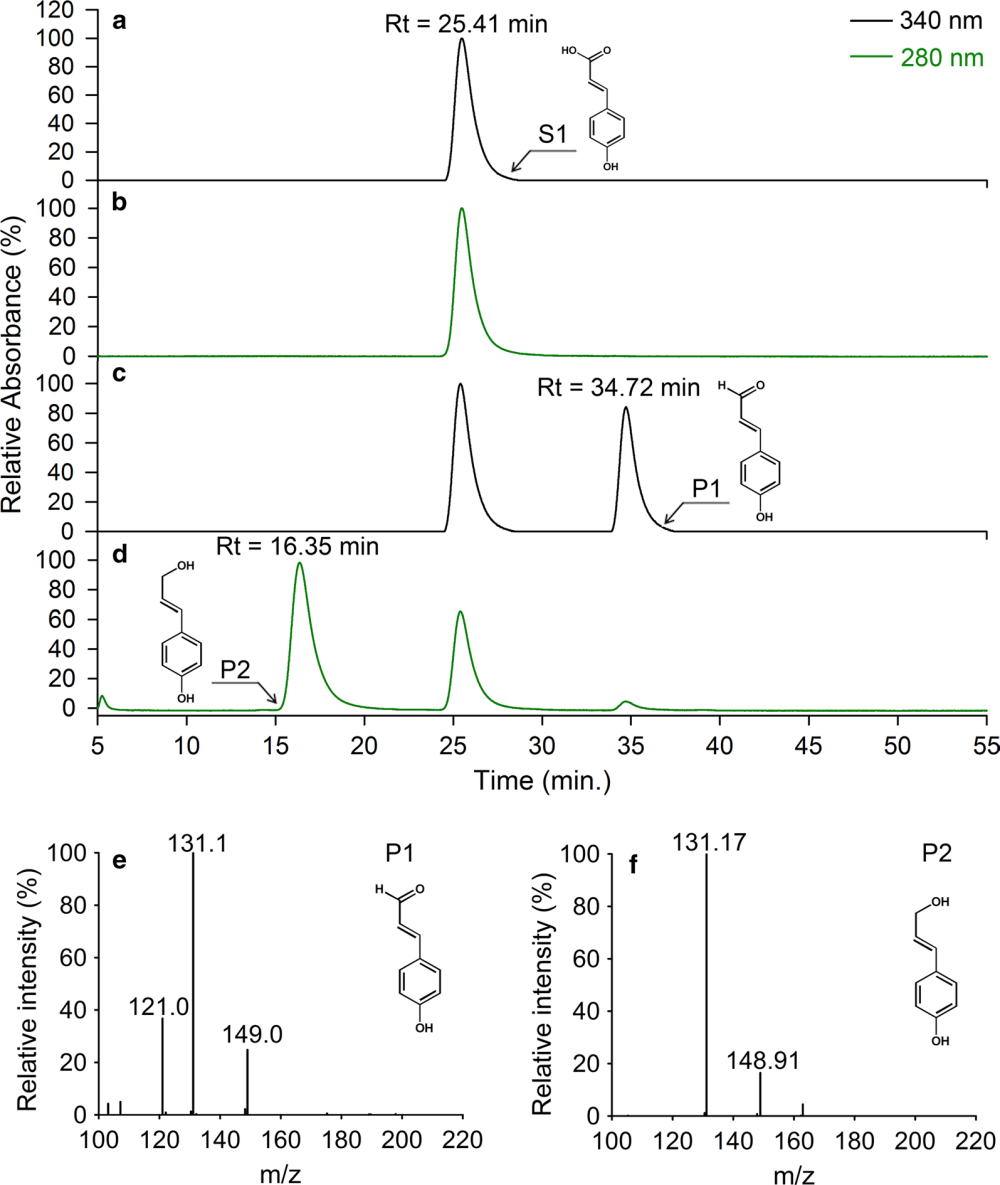
Production of *p*-coumaryl alcohol in immobilized cells. **a** *p*-Coumaric acid (*S1*), detected under 340 nm; **b** *p*-coumaric acid (*S1*), detected under 280 nm; **c** reaction product of *p*-coumaric acid, detected under 340 nm (*P1*); **d** reaction product of *p*-coumaric acid, detected under 280 nm (*P2*); **e** MS/MS profile of *p*-coumaraldehyde under the ESI positive scan mode (*P1*); **f** MS/MS profile of *p*-coumaryl alcohol under the ESI negative scan mode (*P2*)

**Fig. 4:**
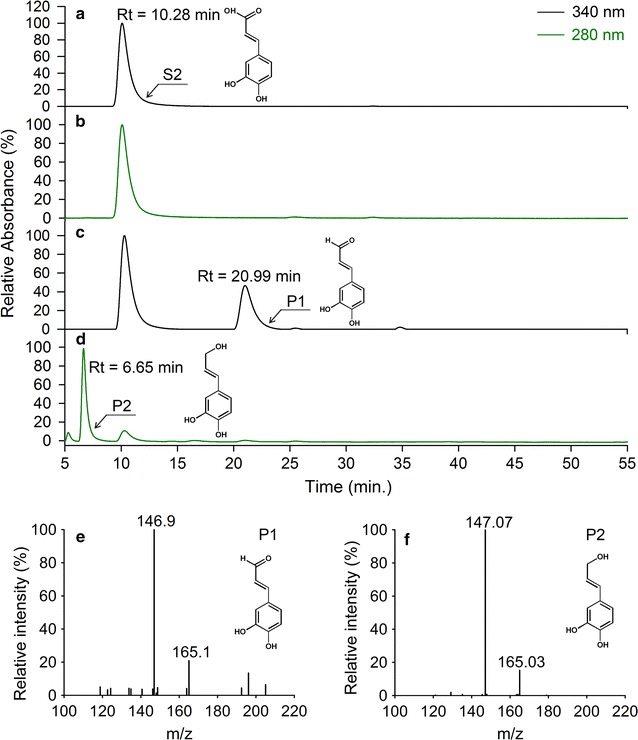
Production of caffeyl alcohol in immobilized cells. **a** Caffeic acid (*S2*), detected under 340 nm; **b** caffeic acid (*S2*), detected under 280 nm; **c** reaction product of caffeic acid, detected under 340 nm (*P1*); **d** reaction product of caffeic acid, detected under 280 nm (*P2*); **e** MS/MS profile of caffeldehyde under the ESI positive scan mode (*P1*); **f** MS/MS profile of caffeyl alcohol under the ESI negative scan mode (*P2*)

**Fig. 5:**
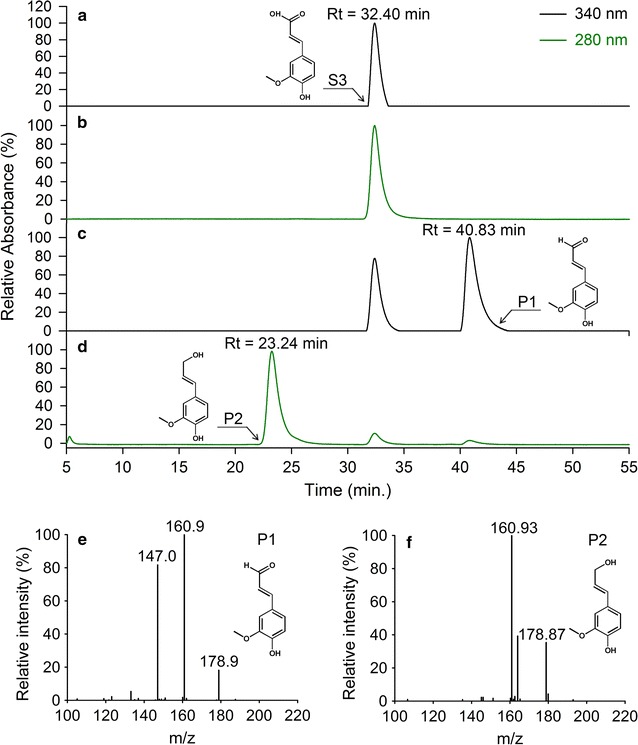
Production of coniferyl alcohol in immobilized cells. **a** Ferulic acid (*S3*), detected under 340 nm; **b** ferulic acid (*S3*), detected under 280 nm; **c** reaction product of ferulic acid, detected under 340 nm (*P1*); **d** reaction product of ferulic acid, detected under 280 nm (*P2*); **e** MS/MS profile of coniferaldehyde under the ESI positive scan mode (P1); **f** MS/MS profile of coniferyl alcohol under the ESI negative scan mode (*P2*)

The immobilized whole cell pellets are displayed in Fig. 2. To examine the feasibility of the immobilized whole-cell system in the biosynthesis of 4-hydroxycinnamyl alcohols, a group of biotransformation experiments were performed with the substrate concentration of 1 mM. The biocatalysis process of three phenylpropanoic acids including *p*-coumaric acid, caffeic acid and ferulic acid were analyzed, and the HPLC–MS/MS chromatograms of these phenylpropanoic acids and their corresponding reductive products are presented (Figs. 3, 4 and 5).

As shown in Fig. 3, the chromatographic peak eluting at 25.41 min was *p*-coumaric acid, and two new peaks appeared under different monitoring wavelengths compared with those in the control group (P1 and P2). To determine whether the unknown chromatographic peaks were the target products, the mass spectra of P1 and P2 were also acquired and analyzed. As demonstrated in Fig. 3e, the molecular mass of P1 was 148 MW (m/z 149 [M+H]^+^), indicating the presence of *p*-coumaraldehyde according to our previous study [34]. The molecular mass of P2 was 150 MW (m/z 149 [M−H]^−^), 2 MW more than the predicted molecular mass of P1, which corresponded with the reduction of an aldehyde group into an alcohol group (Fig. 3f). Based on the retention time and molecular weight of the product, we found that *p*-coumaric acid was converted into *p*-coumaraldehyde and *p*-coumaryl alcohol by the enzyme-catalyzed reduction.

Biotransformation of caffeic acid by immobilized *E. coli* strains resulted in a new product (P1) with an HPLC retention time and molecular mass (164 MW) identical to that of caffealdehyde (Fig. 4c, e), and an unknown chromatographic peak (P2) at 6.65 min. For unknown products, the base peak in the mass spectrum of P2 was m/z 165 under the ESI negative ion scan mode. Its high signal-to noise ratio indicated that the characteristic ion (m/z 147), which was the characteristic fragment of caffeyl alcohol, was clearly observable (Fig. 4f). As expected, the caffeic acid converted into caffeyl alcohol via the new metabolic system.

Similarly, the immobilized *E. coli* produced coniferyl alcohol when ferulic acid was added in the reaction system (Fig. 5). Figure 5d shows a new peak (Rt = 23.24 min) corresponding to coniferyl alcohol, compared with that of the standard solution. The MS/MS spectrum of the reaction product P2 matched the authentic coniferyl alcohol (Fig. 5f). Previous studies showed that 4CL1 from *P. tomentosa* were unable to catalyze the reduction of sinapic acid [36]. Therefore, with immobilized *E. coli* acting as the biocatalyst and its involvement in the biotransformation process, all target products except for sinapinic alcohol were successfully produced. These findings verified that this technique was feasible and reproducible.

### Effects of various culture conditions on the biotransformation capacity

To obtain a higher molar yield of 4-hydroxycinnamyl alcohols, the effects of the pH values and reaction temperatures were investigated. The concentration of coniferyl alcohol produced by dual-cell immobilization system under different conditions was calculated and compared.

#### Effects of the initial pH value on biotransformation capacity

**Fig. 6:**
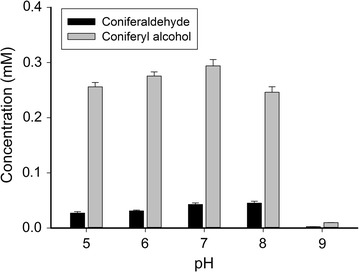
Effects of the initial pH value on biotransformation capacity. The immobilized whole cells were cultivated in LB medium with 1 mM ferulic acid at 30 °C for 10 h. The LB medium was adjusted to pH 5, 6, 7, 8 and 9, respectively. *Error bars* indicate mean values ± SD from three independent experiments

Three enzymes exist in the lignin biosynthesis pathway for the production of 4-hydroxycinnamyl alcohols: 4CL1, CCR and CAD. Thus, it is necessary to find the optimum pH for the reduction reaction with immobilized whole-cells. The effects of the pH values ranging from 5 to 9 are shown in Fig. 6. The results showed that the production of coniferyl alcohol increased from about 87 to 100% as pH value increased from 5 to 7, then declined dramatically with pH values greater than 7. This phenomenon probably caused by the changed charge interactions between multiple proteins and reaction substrates due to varying buffer pH during biotransformation. Therefore, the optimal pH value for biotransformation reaction was pH 7.

#### Effects of the initial temperature on biotransformation capacity

**Fig. 7:**
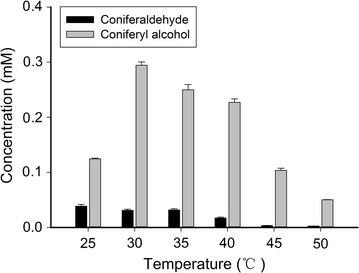
Effects of the initial temperature on biotransformation capacity. The immobilized whole cells were cultivated in LB medium with 1 mM ferulic acid at pH 7 for 10 h. The experiments were performed at temperatures 25, 30, 35, 40, 45 and 50 °C respectively. *Error bars* indicate mean values ± SD from three independent experiments

Temperature affects not only the stability and activity of a biocatalyst, but also the reaction equilibrium. To study the effect of the reaction temperature on the biotransformation capacity, a series of tests were conducted in the temperature range of 25–50 °C. As shown in Fig. 7, temperature played an important role in the biotransformation of ferulic acid. A dramatic increase in coniferyl alcohol accumulation was recorded as the temperature increased to 30 °C, and then it decreased. This finding is consistent with a previous report demonstrating that the optimal temperature for CAD catalysis was 30 °C [35]. Consequently, pH 7.0 and 30 °C were chosen as the best parameters under the experimental conditions, and this value was used in the following experiments.

### Quantitative analysis of 4-hydroxycinnamyl alcohols

**Fig. 8:**
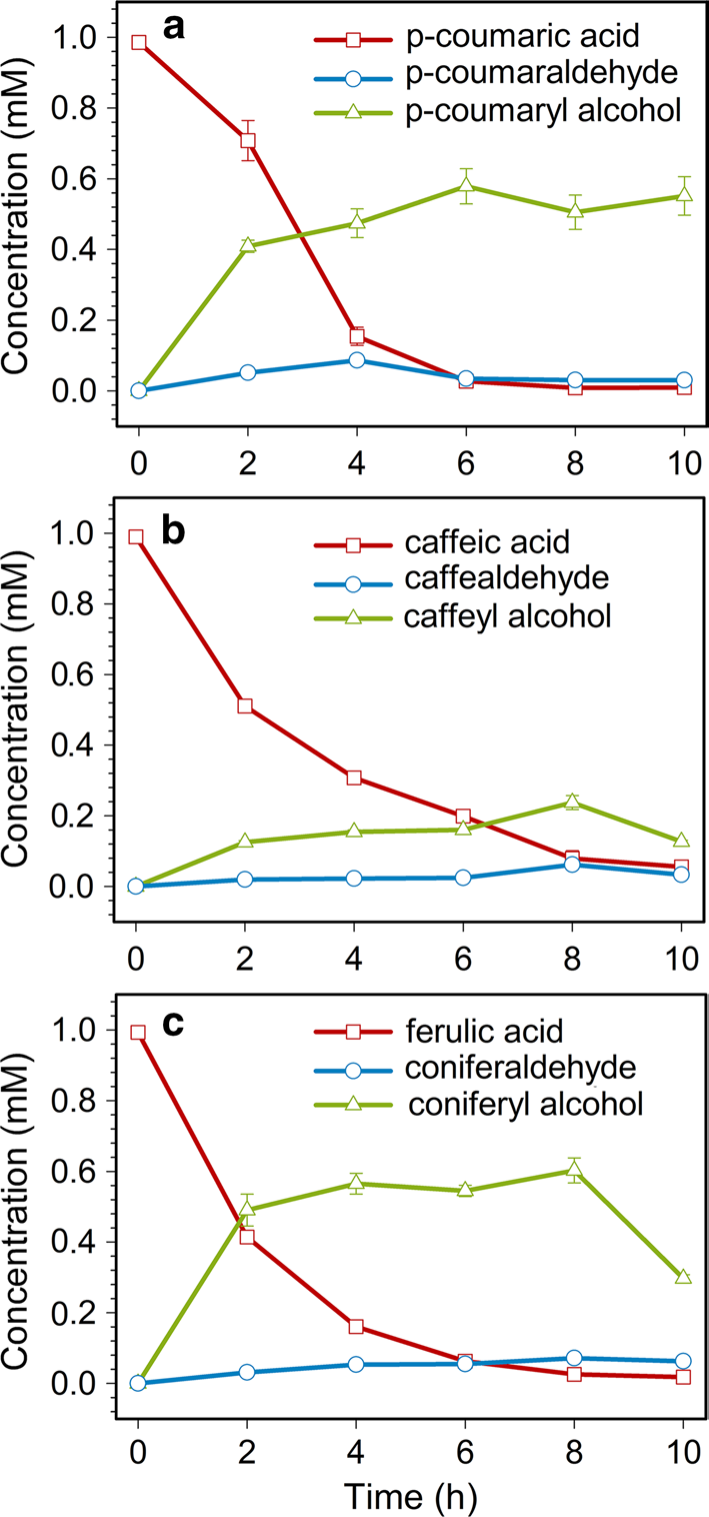
Time-course profiles of 4-hydroxycinnamyl alcohols production. **a** Production of *p*-coumaryl alcohol and consumption of *p*-coumaric acid in *E. coli* strains M15–4CL1–CCR and M15–CAD; **b** production of caffeyl alcohol production and consumption of caffeic acid in *E. coli* strains M15–4CL1–CCR and M15–CAD; **c** production of coniferyl alcohol and consumption of ferulic acid in *E. coli* strains M15–4CL1–CCR and M15–CAD. *Error bars* indicate mean values ± SD from three independent experiments

To evaluate the capability of the dual-cell immobilization system in the biotransformation of phenylpropanoic acids as the substrates, the time-course profiles of 4-hydroxycinnamyl alcohols production were investigated (Fig. 8). Sinapaldehyde was used as an internal standard for an accurate quantitative analysis of the aromatic compounds in our samples. One hundred ng of sinapaldehyde was added to the extract prior to extraction and purification. Following SPE purification and HPLC–PDA–ESI–MSn detection, the peak area ratio of the metabolites to the internal standard, multiplied by the amount of internal standard, provided the quantitative results.

Under the optimized conditions described above, the processes for the production of alcohols were monitored over 10 h. Over this period of time, three exogenous acids gradually reduced and were accompanied by the formation of aldehydes and alcohols. The amount of intermediate aldehydes was low at all times and had no significant changes. The maximum amount of *p*-coumaryl alcohol was produced at 6 h (0.04 mM of *p*-coumaraldehyde and 0.58 mM of *p*-coumaryl alcohol). At 6 h, the amount of *p*-coumaraldehyde declined, but the amount of *p*-coumaryl alcohol reached a maximum (Fig. 8a). The *p*-coumaric acid immediately converted to *p*-coumaraldehyde, and subsequently converted to *p*-coumaryl alcohol within 6 h. Figure 8b shows that caffeic acid was rapidly consumed in the first 8 h, and caffeyl alcohol started to accumulate in the same frame of time. Approximately 0.06 mM of caffeldehyde and 0.24 mM of caffeyl alcohol were produced at 8 h. It seems that some of the caffeic acid is being converted to something other than caffealdehyde or caffeyl alcohol. But the exact mechanism for the consumption of caffeic acid is not fully understood and requires further research. Under these optimum conditions, ferulic acid was completely degraded after 8 h and the concentrations of coniferaldehyde and coniferyl alcohol rose to 0.07 and 0.60 mM, respectively (Fig. 8c). Eight hours later, however, the concentrations of caffeyl alcohol and coniferyl alcohol decreased, which was likely from the alcohol consumption by the microorganism undergoing carbon starvation (Fig. 8b, c). In total, 0.58 mM *p*-coumaryl alcohols, 0.24 mM caffeyl alcohols and 0.60 mM coniferyl alcohols were produced. The conversion ratio and molar yield were calculated and presented in Table 3. Our results indicated that the immobilized whole cells of two recombinant *E. coli* were sufficiently active to convert diverse phenylpropanoic acids to 4**-**hydroxycinnamyl alcohols.

**Table 3:** The molar yield of 4-hydroxycinnamyl alcohols and the corresponding conversion ratio of phenylpropanoic acids

Substrate	Time (h)	Conversion ratio (%)	Product	Molar yield (%)
*p*-Coumaric acid	6	97 ± 1	*p*-Coumaryl alcohol	58 ± 5
Caffeic acid	8	92 ± 2	Caffeyl alcohol	24 ± 2
Ferulic acid	8	97 ± 1	Coniferyl alcohol	60 ± 4

## Discussion

Biotransformations with immobilized whole cells lead to the application of biological steps to chemocatalysis, which makes the whole process more effective. Some immobilized microorganism technologies and materials have been investigated for their high value-added product synthesis [21, 37]. However, most studies found that whole-cell immobilization systems were generally comprised of a single strain. The advantage of using dual-cell immobilization systems for industrial purposes is their ability to combine complimentary metabolic pathways into single functional communities. A multi-strains immobilization system in which has the activities of three enzymes, 4CL1, CCR and CAD, has, to the best of our knowledge, yet to be reported. As such, in this study, we developed a simple and innovative dual-cell immobilization system using the key enzymes in the lignin biosynthesis pathway as the catalyst to produce sequential reactions. We previously reported that the production of 4-hydroxycinnamaldehydes using free whole cells of *E. coli* overexpressing the artificially fused bi-functional enzyme 4CL1–CCR. The goal of this study was to produce 4**-**hydroxycinnamyl alcohols and simplify the production process using multi-strains immobilization to directly convert phenylpropanoic acids to 4**-**hydroxycinnamyl alcohols.

When the immobilized engineered *E. coli* strains M15–4CL1–CCR and M15–CAD were cultured in the media supplemented with *p*-coumaric acid, caffeic acid and ferulic acid, the characteristic spectra of *p*-coumaryl alcohol, caffeyl alcohol and coniferyl alcohol appeared, which confirmed that the multi-strains immobilization system successfully catalyzed the sequential conversions (Figs. 3, 4 and 5). This study used *E. coli* cells expressing bi-functional fusion enzymes instead of cells expressing 4CL1 and CCR, because the intermediate products, hydroxyphenylacetyl-CoA thioesters, were unstable, and more importantly, they could not cross the cell membrane [38]. Clearly, the end products freely passed through the cellular membrane during the biotransformation process. And, from the HPLC spectra, we observed that the reduction products were pure, which greatly simplify the purification steps and reduce the cost of products purification. Results for the biotransformation experiments showed that the immobilized whole cell was a promising biocatalyst and a novel method for the synthesis of natural products.

The calcium alginate entrapment method is the most widely used immobilization technology, which has the advantages of fast curing, low toxicity, chemical and biological stability, high cell density and low cost. Therefore, it is very suitable for the immobilization of microbial cells. Pawar et al. demonstrated that 2% was the optimal level of a sodium alginate solution for the immobilization of *E. coli* cells [39]. This solution allowed a balance between the support stability and the accessible nutrients, which lead to high levels of enzyme production. Our study showed that the pH values and reaction temperatures significantly affected the efficiency and applicability of this system (Figs. 6 and 7). Variation in buffer pH and temperature may not only influence the selectivity and activity of the enzymes, but also the regeneration of the coenzyme present in the microbial cells, which in turns affects the biotransformation [40]. Temperature may affect the diffusion of the substrates, intermediates and products into and out of the cells as well. Obviously, the optimal buffer pH and temperature for the biosynthesis of coniferyl alcohol were 7.0 and 30 °C, respectively.

During the biotransformation process, three exogenous acids gradually reduced and were accompanied by the formation of alcohols without cofactor supplements. As shown in Fig. 8, during the first few hours following the biotransformation of phenylpropanoic acids, the accumulation of 4**-**hydroxycinnamyl alcohols increased. However, as the reaction time continued, the productivity decreased. Thus, it was necessary to halt the bioconversion reaction and extract the metabolites when the target product ceased to increase. This phenomenon may have been caused by the loss of alcohols to the headspace, which indicated that we underestimated our reported production values [41]. Another possibility was that the alcohol transformed to other secondary metabolites by *E. coli* undergoing carbon starvation, which had a negative effect on product accumulation [24, 42]. The exact mechanism for the consumption of alcohol is not fully understood and requires further research.

The substrate preference of the enzymes varied considerably. Previous studies found that 4CL, CCR and CAD from *P. tomentosa* preferred *p*-coumaric acid, feruloyl-CoA and coniferaldehyde [31, 35, 43]. The fusion enzyme 4CL–CCR exhibited the highest affinity to *p*-coumaric acid, followed by ferulic acid and caffeic acid [34]. Our multi-enzyme system retained the enzymatic activity of its individual native enzymes, but its substrate preference varied. Under the combined influence of the fusion enzymes 4CL–CCR and CAD, ferulic acid had the highest conversion ratio during the biotransformation, followed by *p*-coumaric acid, with caffeic acid being the slowest. These enzyme results, along with the yields, showed that ferulic acid was the most favorable substrate for this multi-enzyme reaction system (Table 3).

We also established a highly sensitive and selective HPLC–PDA–ESI–MSn method to identify and quantify the metabolites. The method included crude extraction with ethyl acetate, pre-purification with a SPE cartridge, separation by HPLC, detection with a PDA detector and verification by an ESI–MSn system. With our method, a total of 10 types of compounds were separated in a single injection within 55 min, because the phenylpropanoic acids, 4-hydroxycinnamaldehydes and 4-hydroxycinnamyl alcohols in the same class possessed extremely similar structures, which lead to similar behaviors during the chromatographic separation process (Table 1). Meanwhile, we used mass spectrometry to obtain the fragment information to identify the 4-hydroxycinnamyl alcohols (Table 2). We completed the dada about these aromatic polymers by combining the findings from our previous study with the mass spectrometry results from this study [34]. This method was a rapid and reliable way to conduct a qualitative and quantitative analysis of phenylpropanoic acids, 4-hydroxycinnamaldehydes and 4-hydroxycinnamyl alcohols.

## Conclusions

In this study, we developed a three-step biocatalytic cascade reaction for the production of 4**-**hydroxycinnamyl alcohols from inexpensive phenylpropanoic acids. Two recombinant *E. coli* strains, including M15–4CL1–CCR and M15–CAD whole cells, were immobilized and applied to the catalytic conversion. Under the optimized conditions of a pH of 7.0 and a temperature of 30 °C, the molar yield of the *p*-coumaryl alcohol, the caffeyl alcohol and the coniferyl alcohol was around 58, 24 and 60%, respectively. Moreover, this study introduced a highly sensitive and selective method to characterize these aromatic polymers by HPLC–PDA–ESI–MSn. To the best of our knowledge, this study was the first to immobilize whole cells of recombinant *E. coli* strains to form 4-hydroxycinnamyl alcohols. Overall, this study offers a simple and environmental-friendly way to architect a dual-cell immobilization system, and a promising method for biosynthetic and industrial processes.

## Methods

### Microorganism and culture condition

Two previously genetically engineered *E. coli* strains M15–4CL1–CCR and M15–CAD were used in this study [34, 35]. The strains were cultured in Luria-Bertani (LB) medium containing 100 μg/ml ampicillin and 25 μg/ml kanamycin and grown at 37 °C. Protein expression was induced with 0.4 mM isopropyl-β-D-thiogalactoside (IPTG) when cell optical density (OD_600_) reached 0.6. The incubation of *E. coli* strain M15–4CL1–CCR was continued for another 8 h at 28 °C and *E. coli* strain M15–CAD was continued for another 4 h at 37 °C.

### Cell immobilization

*Escherichia coli* cells were immobilized by entrapment in calcium alginate gel. The proportion of each cell type was equal. About 2 g (wet weight) of bacterial cell pellets were harvested by centrifugation at 4000 rpm for 15 min. The cells were resuspended and washed twice with 10 mL of 0.9% NaCl solution. Subsequently, the bacterial cell slurry (whole cells) was mixed with 50 mL sodium alginate buffer containing 1 g sodium alginate to form a 2% sodium alginate solution. The solution was dropped into a 2% ice-cold calcium chloride solution to form beads. The beads were maintained in calcium chloride solution at 4 °C for 12 h to ensure complete gelification.

### Production of 4-hydroxycinnamyl alcohols in immobilized *E. coli*

The immobilized whole cells were used for the bioconversion of three kinds of phenylpropanoic acids. *p*-Coumaric acid, caffeic acid and ferulic acid were used as substrates and directly added into the culture medium, with a final concentration of 1 mM, respectively. There was no additional cofactor added during the biotransformation process. The production of 4-hydroxycinnamyl alcohols was carried out at 30 °C with agitation at 200 rpm for 10 h.

In order to investigate the effects of the initial pH value on biotransformation capacity, the LB medium was adjusted with HCl or NaOH to pH 5, 6, 7, 8 and 9, respectively.

In order to study the effects of the initial temperature on biotransformation capacity, experiments were performed at temperatures 25, 30, 35, 40, 45, 50 °C.

### Extraction and purification of metabolites

To identify and quantify of the metabolites, 5 ml culture samples were collected and put into a 50 ml centrifuge tube. Sinapaldehyde was added to the centrifuge tube as an internal standard, which serves to correct for random and systematic errors throughout the entire method. Then the samples containing the internal standard were extracted three times with an equal volume of ethyl acetate. The organic phase was collected and concentrated under nitrogen.

The residue was dissolved in 2 ml 5% (V/V) methanol and applied to an Oasis MCX column that had been pre-conditioned with 5 ml methanol and 5 ml acetic acid. After the sample had been loaded, the column was washed with 5 ml 0.1 M acetic acid, the target metabolites were eluted with 5 ml of 0.1 M acetic acid in 40% (V/V) acetonitrile. The eluate was evaporated under vacuum. The residue was redissolved in 200 μl of 50% (V/V) methanol, and ultral-filtered through a micro-filter (4 mm, 0.25 μm) before analysis. Ten microliter aliquots of the filtrate were detected by HPLC–PDA–ESI–MSn.

### Detection and characterization of metabolites by HPLC–PDA–ESI –MSn

The metabolites were separated by an HPLC system (Thermo Finnigan, Waltham, MA, USA) using 0.1% (V/V) formic acid in water (buffer A) and 100% acetonitrile (buffer B) as mobile phases at a flow rate of 0.15 ml/min. The HPLC separation was carried out using a reversed-phase column (ZORBAX 300SB-C18, 2.1 × 150 mm, 3.5 μm; Agilent, Santa Clara, CA, USA). The gradient profile was 8% B for 2 min, increased to 20% B in 38 min, then to 100% B in 12 min and maintained for 10 min, and decreased to 8% B in 2 min and maintained for 10 min. The acquisition time was 55 min and delay time was 5 min per spectrum. The metabolites were detected by monitoring the absorption at 280 and 340 nm.

The metabolites were identified using an ion trap mass spectrometer (LCQ DECA XP MAX) coupled with an ESI source (Thermo Finnigan). The MS parameters for analysis were: capillary temperature 280 °C, spray voltage 4.5 kV, sheath gas (nitrogen) flow rate 40 arb and aux/sweep gas (nitrogen) flow rate 10 arb. Collision energy and other tune parameters were optimized for dissociation of parent ions into product ions for each metabolite. The mass spectrometer was acquired in data-dependent MS/MS mode: each full MS scan (in the range 100–220 m/z) was followed by four MS/MS of selected ions including substrate, intermediate product, end product and internal standard.

### Quantification of 4-hydroxycinnamyl alcohols

Data processing was performed in Xcalibur 2.1 (Thermo Finnigan). The external standards and internal standard were prepared for quantification purposes in order to create calibration curves and account for variable losses during the preparation steps including sample extraction and purification, chromatography and MS detection. Sinapaldehyde is an ideal internal standard which possesses characteristics similar to those of aromatic compounds and does not interfere with the target analytes. Calibration curves were generated by adding defined amounts of compound and calculated by plotting the peak area (Table 1). Each analysis was performed in triplicate. Because *p*-coumaryl alcohol and caffeyl alcohol are not commercially available, we used coniferyl alcohol to generate an external standard curve for quantitative analysis of the reaction products. The conversion ratio and molar yield were calculated using the following equation:

$${\text{Conversion ratio}}\;\left( \% \right) = \left( {C_{S1} { - }C_{S2} } \right)/C_{S1} \times 100\%$$

$${\text{Molar yield}}\;\left( \% \right) = C_{P} /C_{S1} \times 100\%$$

wherein *C*_*S1*_ is the initial concentration of substrate, *C*_*S2*_ is the equilibrium concentration of substrate, *C*_*P*_ is the equilibrium concentration of product.

## Abbreviations

4CL1: 4-coumaric acid: coenzyme A ligase
CCR: cinnamoyl coenzyme A reductase
CAD: cinnamyl alcohol dehydrogenase
HPLC–PDA–ESI–MSn: high performance liquid chromatography–photo diode array–electrospray ionization–ion trap mass spectrometry
ESI–Ion trap–MS: electrospray ionization ion trap mass spectrometry
SRM: selected reaction monitoring
MS/MS: tandem mass spectrometry
PS: pinoresinol synthase
PLR: pinoresinol reductase
ATP: adenosine triphosphate
CoA: coenzyme A
NADPH: reduced form of nicotinamide adenine dinucleotide phosphate
LB: Luria-Bertani
IPTG: isopropyl-β-d-thiogalactoside
